# Antigenicity of peptides comprising the immunosuppressive domain of the retroviral envelope glycoprotein

**DOI:** 10.12688/wellcomeopenres.10269.2

**Published:** 2017-02-21

**Authors:** Bryony Jenkins, Urszula Eksmond, George Young, George Kassiotis

**Affiliations:** 1 The Francis Crick Institute, London, UK; 2 Department of Medicine, Faculty of Medicine, Imperial College London, London, UK

**Keywords:** Immunosuppressive domain, retroviral envelope, T cell response, T cell receptor, peptide-MHC II complex

## Abstract

To achieve persistent infection of the host, viruses often subvert or suppress host immunity through mechanisms that are not entirely understood. The envelope glycoprotein of several retroviruses is thought to possess potent immunosuppressive activity, mapped to a 17-amino acid residue conserved domain. Synthetic peptides corresponding to this immunosuppressive domain can inhibit lymphocyte activation, whereas mutation of key domain residues can increase the lymphocyte response to linked antigenic epitopes. Using three T cell receptors (TCRs) of defined specificity, we examine the effect of the immunosuppressive domain on the T cell response to their respective antigenic peptides. We find that fusion of a T cell epitope to the immunosuppressive domain can greatly modulate its potency. However, the effects heavily depend on the particular combination of TCR and peptide-major histocompatibility complex class II (pMHC II), and are mimicked by sequence-scrambled peptides of similar length, suggesting they operate at the level of pMHC formation or TCR-pMHC interaction. These results offer an alternative explanation for the immunogenicity of T cell epitopes comprising the putative immunosuppressive domain, which is more consistent with an effect on peptide antigenicity than true immunosuppressive activity.

## Introduction

Several studies over the last three decades have provided evidence to suggest that the envelope glycoprotein of certain retroviruses is immunosuppressive *in vitro* and *in vivo* ( Cianciolo *et al.*, 1985; de Parseval *et al.*, 2001; Denner, 2014; Dupressoir *et al.*, 2012; Haraguchi *et al.*, 1997; Kudo-Saito *et al.*, 2014; Mangeney *et al.*, 2007; Schlecht-Louf *et al.*, 2010). Although the precise molecular mechanism remains unresolved, immunosuppressive activity has been pinpointed at a short region of the transmembrane polypeptide that, together with the surface unit (SU), constitutes one part of the trimeric envelope glycoprotein ( Cianciolo *et al.*, 1985; Dupressoir *et al.*, 2012; Schlecht-Louf *et al.*, 2010). For example, synthetic peptides corresponding to a region of 17-amino acid residues from the murine leukemia virus (MLV) envelope glycoprotein inhibit immune function in a variety of assays. These assays measure distinct aspects of immune responsiveness. Some measure non-specific lymphocyte or myeloid cell activation in response to mitogens or other generic stimuli ( Denner, 2014; Denner *et al.*, 1996; Haraguchi *et al.*, 1993; Haraguchi *et al.*, 1995; Mitani *et al.*, 1987; Tolosa *et al.*, 2012). Others measure antigen-specific T and B cell responses to immunization with synthetic peptides, recombinant envelope domains, and to infection with MLVs ( Morozov *et al.*, 2012; Schlecht-Louf *et al.*, 2014; Schlecht-Louf *et al.*, 2010). Use of different assays, many of which are multilayered, has hindered direct comparison of results from different studies, or definition of the specific step in immune function that may be suppressed. Here, we examine the properties of T cell epitopes linked to the immunosuppressive domain and provide an alternative interpretation for the effects.

## Materials and methods

### Mice

All animal experiments were approved by the Ethical Committee of the Francis Crick Institute, and conducted according to local guidelines and UK Home Office regulations under the Animals Scientific Procedures Act 1986. Inbred C57BL/6J and BALB/cJ mice were originally obtained from The Jackson Laboratory (Bar Harbor, ME, USA) and subsequently maintained at the Francis Crick Institute’s animal facilities, under specific pathogen-free conditions. Male or female mice, between 8 and 12 weeks of age, were used for the isolation of macrophages. T cell receptor (TCR)-transgenic OT-II ( Barnden *et al.*, 1998) and DO11.10 ( Murphy *et al.*, 1990) mice were kept on the C57BL/6J and BALB/cJ genetic backgrounds, respectively, and were additionally crossed to *Rag1 ^-/-^* mice to prevent rearrangement of endogenous TCR loci.

### Macrophages

Macrophages were isolated from the peritoneal cavity of naïve euthanized C57BL/6J or BALB/cJ mice (2–6 mice per experiment). Peritoneal cells (containing ~50% macrophages) were plated in flat-bottomed 96 well-plates at 4×10 ^5^ per well the day before their use, in Iscove's Modified Dulbecco's Medium (IMDM; Sigma-Aldrich, St. Louis, MO, USA) supplemented with 5% fetal bovine serum (Gibco, Thermo Fisher Scientific, Waltham, MA USA), 2mM L-glutamine, 100U penicillin and 0.1mg/ml streptomycin, and cultured at 37°C in a 5% CO _2_ atmosphere. Following overnight culture, macrophages were enriched by the removal of non-adherent cells, and were used for the assays.

### T cell hybridomas

The env _124–138_-reactive H5 and H18 CD4 ^+^ T cell hybridomas have been previously described ( Young *et al.*, 2012). The ova _323–339_-reactive OT-II and DO11.10 hybridomas were generated by fusion of ova _323–339_-stimulated primary splenic CD4 ^+^ T cells from single TCR-transgenic OT-II and DO11.10 mice, respectively, with TCRαβ-negative BW5147 thymoma cells, using established techniques ( Young *et al.*, 2012). In at least two experiments, primary CD4 ^+^ T cells from TCR-transgenic mice were also used with comparable results.

### Peptides encompassing the immunosuppressive domain

Peptides were synthesized by Insight Biotechnology Ltd., Wembley, UK, at >98% purity. Scrambled sequences represent permutation of the original peptide sequences and were constructed using the peptide manufacturer’s algorithms. All peptides were sufficiently hydrophobic and were dissolved in phosphate-buffered saline prior to use ( Table 1).

**Table 1.** Peptides used in this study. The following peptides were synthetized and used as indicated in the text. The underlined amino acid residues indicate the E561R and A567F double mutation ( Schlecht-Louf *et al.*, 2010). Red coloured sequences correspond to the immunosuppressive peptide.

Peptide name	Sequence
env _124-138_	PLTSLTPRCNTAWNR
env _548-567_	LQNRRGLDLLFLKEGGLCAA
env _548-567:E561R/A567F_	LQNRRGLDLLFLK RGGLCA F
env _124-138_-env _548-567_	PLTSLTPRCNTAWNR LQNRRGLDLLFLKEGGLCAA
env _124-138_-env _548-567:E561R/A567F_	PLTSLTPRCNTAWNR LQNRRGLDLLFLK RGGLCA F
env _124-138_-env _548-567:SCRAMBLED_	PLTSLTPRCNTAWNRAGFAKGLRDGLRQLNELCLL
env _124-138_-env _548-567:E561R/A567F:SCRAMBLED_	PLTSLTPRCNTAWNR FGFAKGLRDGLRQLN RLCLL
env _124-158_	PLTSLTPRCNTAWNRLKLDQVTHKSSEGFYVCPGS
ova _323-339_	ISQAVHAAHAEINEAGR
ova _323-339_-env _548-567_	ISQAVHAAHAEINEAGR LQNRRGLDLLFLKEGGLCAA
ova _323-339_-env _548-567:E561R/A567F_	ISQAVHAAHAEINEAGR LQNRRGLDLLFLK RGGLCA F

### T cell stimulation

T cell hybridomas were stimulated by the indicated peptides presented by primary macrophages. T cell clones restricted by H2-A ^b^ (H5, H18 and OT-II) were stimulated by C57BL/6J-derived macrophages, whereas the H2-A ^d^-restricted clone (DO11.10) was stimulated by BALB/cJ-derived macrophages. Similar results were also obtained when primary splenic B cells or bone-marrow-derived dendritic cells were used for presentation. Approximately, 1.5×10 ^5^ T cells per well were used in flat-bottomed 96 well-plates in RPMI-1640 medium (Sigma-Aldrich, St. Louis, MO, USA) containing 10% fetal calf serum. The following day, T cell activation was assessed by flow cytometric detection of CD69 induction, using a fluorescein isothiocyanate-conjugated monoclonal anti-mouse CD69 antibody (armenian hamster; clone H1.2F3; eBioscience, San Diego, CA, USA; cat. no. 11-0691-85) at 1 in 200 dilution. At least 50,000 cells were acquired on LSRFortessa X-20 or FACSCanto II cytometers (BD Biosciences, San Jose, CA, USA) and analyzed with FlowJo v10 (Tree Star Inc., Ashland, OR, USA). Unstimulated hybridoma cells were included as CD69-negative controls, based on which the gating of CD69-postitive cells was drawn.

### Transcriptional profiling by RNA sequencing

Transcriptional profiles of primary macrophages incubated with the env _124–138_-env _548–567_ and env _124–138_-env _548–567:E561R/A567F_ peptides were obtained by RNA sequencing. Briefly, duplicate cultures of peritoneal macrophages from C57BL/6 mice were stimulated overnight with 10µM concentration of either peptide or were left unstimulated. RNA was then extracted using the RNeasy Mini QIAcube kit (Qiagen, Hilden, Germany) following the manufacturer’s instructions, and subjected to RNA sequencing (GENEWIZ, Inc., South Plainfield, NJ, USA). Reads were assessed for quality and contamination with FastQC v0.11.2 ( http://www.bioinformatics.babraham.ac.uk/projects/fastqc/) and preprocessed with Trimmomatic v0.32 ( http://www.usadellab.org/cms/?page=trimmomatic) to remove the identified adapters, low quality read tails and to filter for length, and were then assessed for differential expression with DESeq2 v1.6.3 ( http://bioconductor.org/packages/release/bioc/html/DESeq.html) within R v3.1.3. Data for all samples were normalized and rlog transformed and were then analyzed using GeneSpring v12.1 GX (Agilent, Santa Clara, CA, USA).

### Statistical analysis

Statistical comparisons were made using SigmaPlot 13.0 (Systat Software Inc., Germany). Parametric comparisons of normally-distributed values that satisfied the variance criteria were made by unpaired Student's t-tests. Data that did not pass the variance test were compared with non-parametric two-tailed Mann-Whitney Rank Sum tests. P<0.05 were considered significant.

## Results and discussion

**Figure 1.**
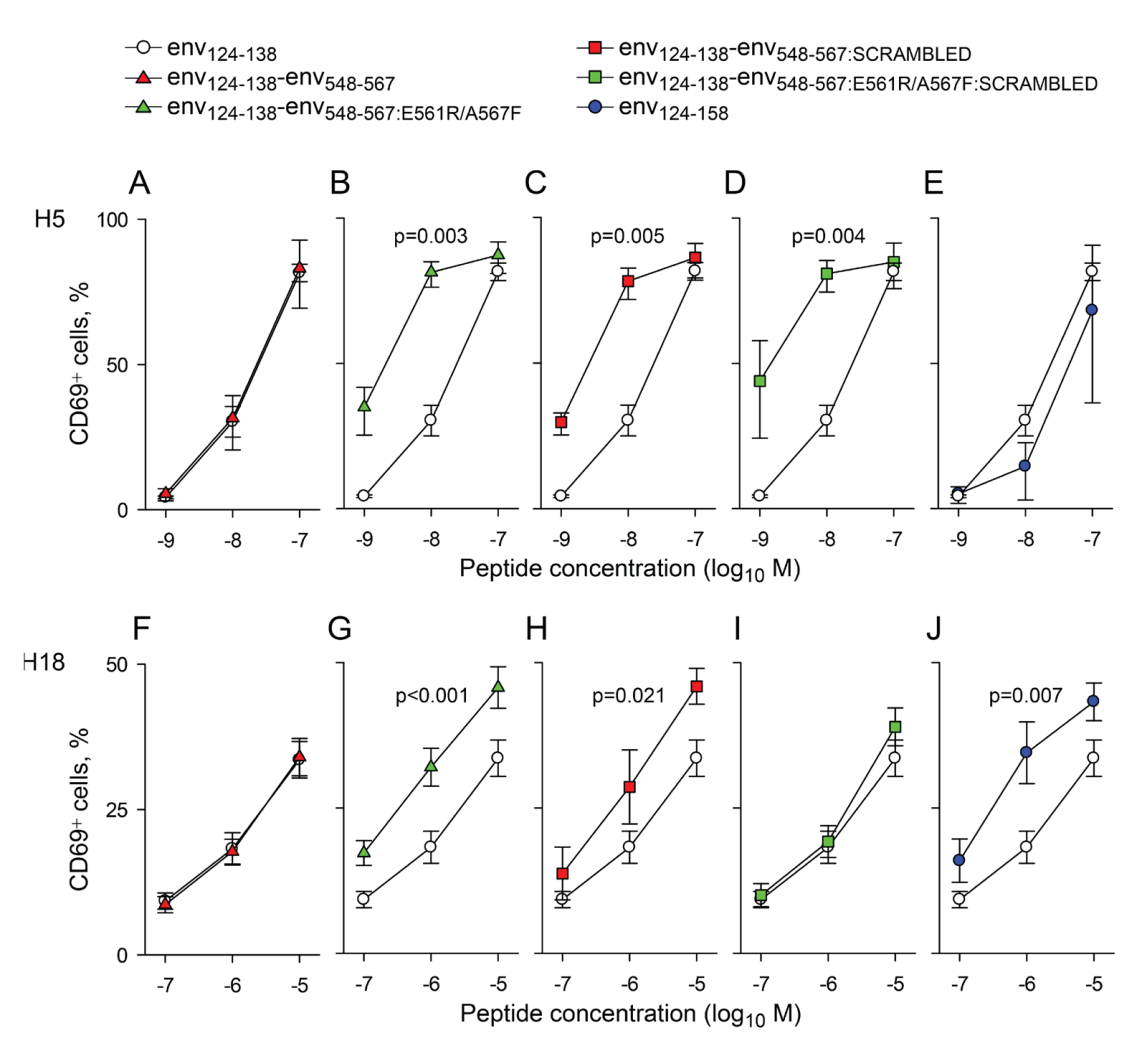
*In vitro* responses of A ^b^-restricted env _124–138_-reactive CD4 ^+^ T cell hybridomas H5 (top row) and H18 (bottom row), to stimulation with the indicated peptides. Plotted are the frequencies of T cells that express CD69 following overnight stimulation. Data are the mean (±SEM) of 2–5 individual experiments.

We used synthetic peptides corresponding to the conserved env _548–567_ region of the Friend MLV envelope precursor gPr80 (LQNRRGLDLLFLKEGGLCAA), which contains the originally described 17-amino acid residue immunosuppressive peptide ( Cianciolo *et al.*, 1985). As controls, we introduced two amino acid substitutions (E561R and A567F), previously shown to abrogate the immunosuppressive activity of this region ( Schlecht-Louf *et al.*, 2010), and also used scrambled sequences for both peptides ( Figure 1). These peptides were tested for their effects on the antigenicity of the H2-A ^b^-restricted env _124–138_ CD4 ^+^ T cell epitope in the SU of F-MLV (PLTSLTPRCNTAWNR). As immunosuppressive peptides have generally been found active only as part of larger polypeptides ( Denner, 2014), we used peptide fusion of the T cell epitope and the immunosuppressive peptide into a single molecule (env _124–138_-env _548–567_). Fusion with the immunosuppressive peptide env _548–567_ had no measureable effect on the ability of the env _124–138_ epitope to stimulate two env _124–138_-specific CD4 ^+^ T cell clones ( Figure 1A and F) that differ in functional avidity ( Young *et al.*, 2012). CD4 ^+^ T cell responses to env _124–138_ were similarly unaffected by the addition in the cultures of the immunosuppressive peptide env _548–567,_ either as a separate entity or fused with the unrelated H2-A ^b^-restricted ova _323–339_ epitope from chicken egg ovalbumin (ISQAVHAAHAEINEAGR) ( Supplementary Figure 1). In contrast, fusion with either the doubly point-mutated ( Figure 1B and G; p=0.003 and p<0.001 for clones H5 and H18, respectively) or sequence-scrambled immunosuppressive peptide ( Figure 1C and H; p=0.005 and p=0.021 for clones H5 and H18, respectively) significantly increased the antigenicity of the env _124–138_ epitope for both clones by ~10-fold. Increased antigenicity of the env _124–138_ epitope as a result of mutations at the two residues or sequence scrambling of the fused immunosuppressive peptide could be interpreted as reversal of immunosuppression due to these modifications. However, comparison with the env _124–138_ epitope on its own revealed that fusion with the index immunosuppressive peptide was in fact neutral ( Figure 1A and F), whereas fusion with the modified immunosuppressive peptides improved antigenicity.

Peptide length is an important contributor to the antigenicity of MHC II-restricted epitopes. Indeed, N-terminal extensions of the env _124–138_ epitope have been shown to increase antigenicity ( Young *et al.*, 2012), in line with findings in other systems ( Carson *et al.*, 1997; Holland *et al.*, 2013). The open structure of the MHC II groove allows presentation of peptides that extend at either end of the core epitope, and these epitope-flanking residues can contribute to T cell stimulation ( Holland *et al.*, 2013). Importantly, the involvement of epitope-flanking residues critically depends on the particular pair of TCR and peptide-MHC II (pMHC) complex, as contact between particular epitope-flanking residues and residues in the TCR is necessary ( Holland *et al.*, 2013). To examine whether the observed effect of the index or modified immunosuppressive peptides was TCR- and peptide sequence-dependent, we used additional fusion peptides. Notably, a peptide that carried the E561R/A567F substitutions and was additionally sequenced-scrambled enhanced antigenicity of the env _124–138_ epitope for the H5 clone (p=0.004), but not the H18 clone ( Figure 1D and I). Moreover, extending the length of the env _124–138_ epitope with the sequence that naturally occurs in the F-MLV SU (env _124–158_) enhanced activation of the H18 clone (p=0.007), but not of the H5 clone ( Figure 1E and J). These two clones share the same TCRβ chain, but use different TCRα chains, which are responsible for the difference in functional avidity ( Young *et al.*, 2012). The disparate behaviour of the two clones in response to the last two fusion peptides demonstrate that changes in antigenicity depend on the particular TCR. This finding argues against general immunosuppression caused by the env _548–567_ peptide.

**Figure 2.**
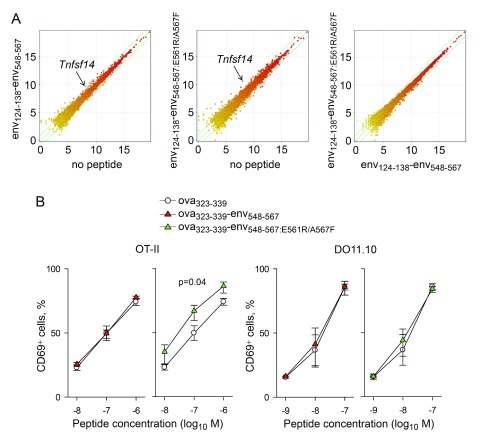
*In vitro* responses of peritoneal macrophages or hybridomas OT-II and DO11.10 to stimulation with the indicated peptides. ( **A**) Transcriptional comparison of primary macrophages incubated overnight in the absence of peptide or in the presence of 10 µM of the indicated peptide. Each dot represents the normalized counts for individual transcripts and green diagonal lines enclose changes in relative transcript abundance of <2-fold in each comparison. ( **B**) *In vitro* responses of A ^b^-restricted and A ^d^-restricted ova _323-339_-reactive CD4 ^+^ T cell hybridomas OT-II and DO11.10, respectively, to stimulation with the indicated peptides. Data are the mean (±SEM) of 2–3 individual experiments.

Also arguing against immunosuppressive ability, env _124–138_ fusion peptides containing the index immunosuppressive peptide or the E561R/A567F mutant induced comparable transcriptional changes to the APCs used in these assays ( Figure 2A). Incubation of primary macrophages with either of the two env _124–138_ fusion peptides altered their transcriptional signature in comparison with the absence of peptide, with *Tnfsf14* (also known as HVEM-L or LIGHT) most strongly induced (between 3-and 4-fold) ( Figure 2A). However, the transcriptional signatures induced by the two peptides were effectively identical ( Figure 2A).

We next examined if our findings with env _124–138_-specific CD4 ^+^ T cell clones extended to other combinations of TCR and pMHC. To this end, we used the OT-II and DO11.10 clones, which react with the same ova _323–339_ epitope presented by H2-A ^b^ and H2-A ^d^, respectively. Fusion of the ova _323–339_ epitope with the env _548–567_ E561R/A567F mutant, but not the index immunosuppressive peptide enhanced the response of the OT-II clone ( Figure 2B) (p=0.04). In contrast, neither peptide fusion affected the response of the DO11.10 clone ( Figure 2B), indicating that effects of the immunosuppressive peptides can be observed in some pMHC combinations or TCR-pMHC pairs, but not others. Thus, this pattern of changes in T cell activation, depending on the particular TCR-pMHC pair, is more consistent with an effect mediated by epitope-flanking residues than genuine immunosuppression. The potential of the immunosuppressive peptide, and modifications thereof, to alter TCR or BCR recognition of linked epitopes should, therefore, be considered when interpreting the antigenicity or immunogenicity of the retroviral envelope glycoprotein.

## Data availability

RNA-sequencing data reported in this paper are available at: www.ebi.ac.uk/arrayexpress/experiments/E-MTAB-5260 (accession number, E-MTAB-5260).

Raw values for T cell responses shown in Figure 1, Figure 2B and Supplementary Figure 1 are available from the Open Science Framework: https://osf.io/8y8h4/; doi, 10.17605/OSF.IO/8Y8H4 ( Kassiotis, 2016).

## Supplementary material

**Supplementary Figure 1: *In vitro* responses of Ab-restricted env124-138-reactive CD4+ T cell hybridoma H18, to stimulation with the indicated combination of peptides.** The X-axis denotes the concentration of the env124–138 peptide. All other peptides were given at constant 10–6 M concentration. Plotted are the frequencies of T cells that express CD69 following overnight stimulation. Data re the mean (±SEM) of 1–6 individual experiments.
