# Supplementary figures and images for: Antigenicity of peptides comprising the immunosuppressive domain of the retroviral envelope glycoprotein
Source: Wellcome Open Res. 2017 Feb 21;1:22. Originally published 2016 Dec 6. [Version 2] doi: 10.12688/wellcomeopenres.10269.2 (PMC5242373; doi:10.12688/wellcomeopenres.10269.2)

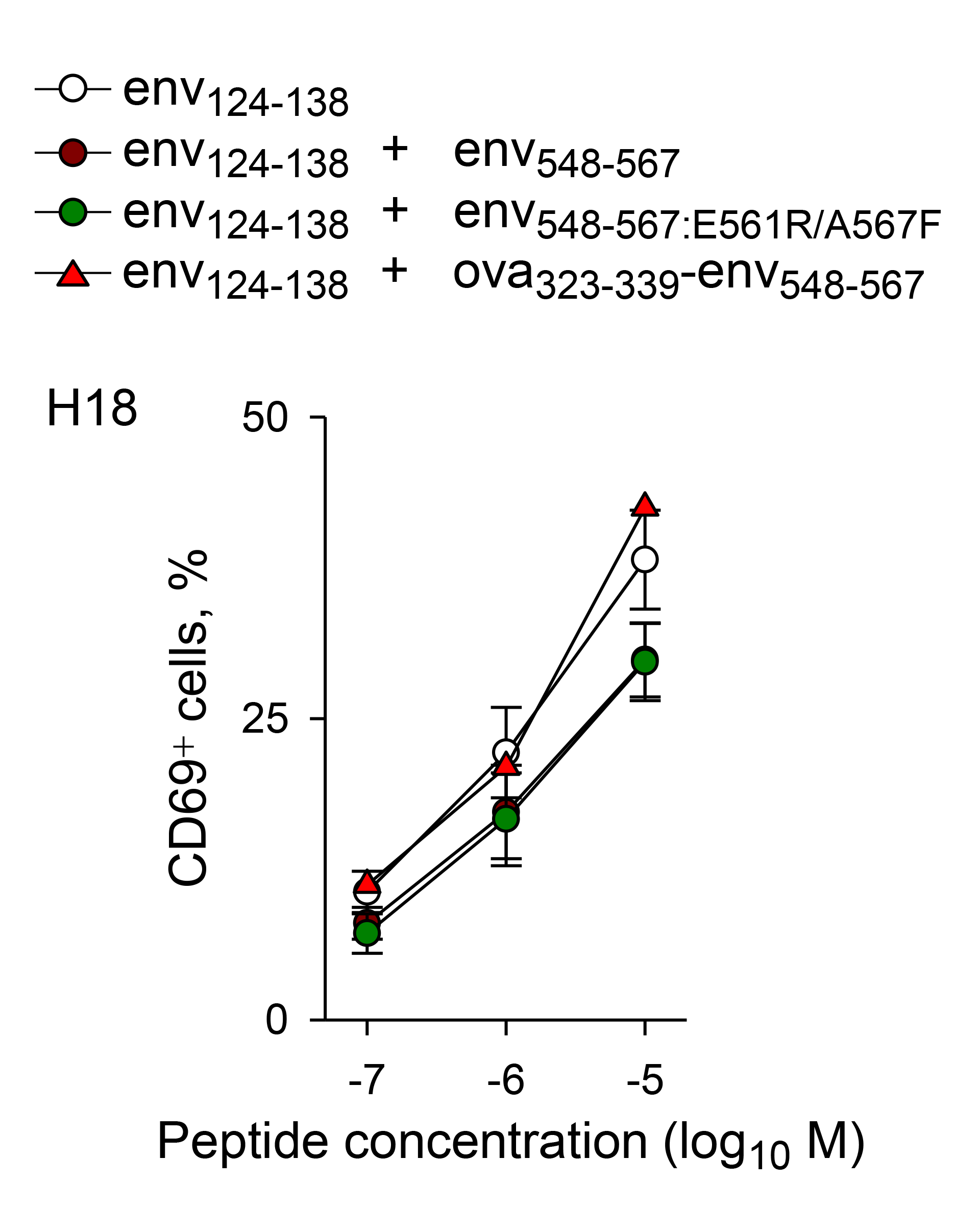

Supplement: Supplementary file 1 [file wellcomeopenres-1-11822-s0000.tgz › 21f5a6fb-a938-4001-832f-68da9736f914.doc]
