# Supplementary material for: Antigenicity of peptides comprising the immunosuppressive domain of the retroviral envelope glycoprotein
Source: Wellcome Open Res. 2017 Feb 21;1:22. Originally published 2016 Dec 6. [Version 2] doi: 10.12688/wellcomeopenres.10269.2 (PMC5242373; doi:10.12688/wellcomeopenres.10269.2)
Supplement: Peer review history

# Peer review: Referee response for version 1

**DOI:** 10.21956/wellcomeopenres.11059.r18509

**Published:** 2017-01-16

**Authors:** Stephanie Gras, Emma Grant, Carine Farenc

**Affiliations:** 1 Infection and Immunity Program and Department of Biochemistry and Molecular Biology, Biomedicine Discovery Institute, Monash University, Clayton, VIC, Australia; 2 Monash University , Clayton, VIC, Australia; 3 Monash Univeristy, Clayton, VIC, Australia

This work gives insights into the immunosuppressive role of the envelope glycoprotein of certain retrovirus. They present the T cell response of 3 TCRs stimulated with different peptides encompassing both the T cell epitope and the immunosuppressive domain. The research note is of interest, clear and well written, and the final conclusion seems appropriate.

**Some section could be developed or clarify:**

1. Abstract: “The effects heavily depend on the particular combination of TCR and peotide-major histocompatibility complex class II, and are mimicked by sequence-scrambled peptides of similar length, suggesting they operate at the level of TCR-pMHC interaction”. The addition/substitution within the peptides could also influence the peptide stability within the MHC-II, or half-life of the pMHC-II complex, and therefore change the T cell stimulation, this should probably be discuss, or acknowledge that the TCR-pMHC-II interactions might not explain everything.
2. Methods. A brief explanation about “scrambled” peptides would be helpful, as would a sentence explaining why peptides were reconstituted in PBS rather then DMSO.
3. Conclusion. I would like a bit more explanation for the unexpected results. As well as maybe considering taking into account the comment from the abstract to re-word the strong conclusion “ *In contrast, neither peptide fusion affected the response of the DO11.10 clone, indicating that effects of the immunosuppressive peptides can be observed in some TCR-pMHC pairs, but not others”.*

 

**Minor comments:**

1. Title: I would suggest changing “comprising” by “within”.
2. Table 1: the shade section is not visible, either the colour needs to be darker or maybe bold the section.
3. The peptides env548-567 and env548-567-E561R-A567F are in Table 1 but don’t seem to be used in the exp? Were they used as control?
4. The env124-138 + ova323-339-env548-567 used in the supplemental figure are not listed in this table.

We have read this submission. We believe that we have an appropriate level of expertise to confirm that it is of an acceptable scientific standard.

# Response

**Published:** 2017-02-14

**Authors:** George Kassiotis

**Affiliations:** The Francis Crick Institute, UK

We wish to thank the Reviewers for their astute and constructive comments. Below is a point-by-point reply and a list of changes we have made to the manuscript in response to these comments:

**Major comments:**

 1.We agree with the Reviewer that peptide alterations could affect the peptide-MHC-II complex (pMHC) itself, not only TCR binding to it. Our definition of TCR-pMHC interaction was meant to be broad enough to include effects on pMHC formation. Obviously, anything that affects pMHC formation or stability will also affect TCR-pMHC interaction, by reducing ligand availability. All these alternatives are on the same side of the argument, the other side being biological activity of the immunosuppressive domain. We have modified this sentence to read: “…at the level of pMHC formation or TCR-pMHC interaction.”

 2. “Scrambled” peptides are permutation of the original peptide sequence. The rationale for using them is that they would retain the physico-chemical characteristics (molecular mass, polarity, acidity, etc), but not any sequence-dependent domain, structure or recognition motif. Practically, these were constructed using the peptide manufacturer’s (GeneScript) algorithms. The peptides were all sufficiently hydrophobic to dissolve in PBS. This was chosen in order to avoid any effect of organic solvents.

 3. Again we agree with the Reviewer and rephrased this sentence to read: “…in some pMHC combinations or TCR-pMHC pairs, but not others”. Given what is known about peptide-flanking residues and their effect on pMHC formation and stability and TCR binding, the results might not be unexpected. In fact, we believe this is the most parsimonious explanation for the effects of the putative immunosuppressive domain, one that needs not invoke any new biological activity or mechanism.

**Minor comments:**

 1. The word “comprising” in this context was meant to be synonymous with “including” or “containing”. The word “within” would have the opposite meaning. The immunosuppressive domain is within the antigenic peptides, not the reverse.

 2. The shading has been replaced with red colour text for enhanced contrast.

 3. These peptides were used for the experiments described in Supplementary figure 1.

 4. The env124-138 and ova323-339-env548-567 peptides were used as separate entities, not as a fusion. They are listed in rows 1 and 10 of Table 1, respectively.

# Peer review: Referee response for version 1

**DOI:** 10.21956/wellcomeopenres.11059.r18343

**Published:** 2016-12-08

**Authors:** Jean-Marie Saint-Remy

**Affiliations:** 1 Center for Molecular and Vascular Biology, University of Leuven, Leuven, Belgium

I think this paper addresses a major issue with consequences for basic research and for the design of new forms of immunotherapy. The concept of “reversal of immunosuppression” is fundamental and stands, luckily enough, in contrast with the overwhelming  view that every bit of immunological studies should be considered as eliciting “active immunosuppression”.

 This paper is therefore novel and susceptible to shed new light in the field.

 Experiments are neatly planned and well described.

 The discussion is well equilibrated.

 I therefore strongly recommend the indexing of these new findings.

I have read this submission. I believe that I have an appropriate level of expertise to confirm that it is of an acceptable scientific standard.
